# P-1783. Blood Metagenomic Next-Generation Sequencing and its Impact on Antimicrobial Management

**DOI:** 10.1093/ofid/ofaf695.1952

**Published:** 2026-01-11

**Authors:** Chad Hinkle, Christopher Lehmann, Vera Tesic, Angelica Moran

**Affiliations:** University of Chicago Medicine, Chicago, IL; University of Chicago, Chicago, Illinois; University of Chicago, Department of Pathology, Chicago, Illinois; University of Chicago, Chicago, Illinois

## Abstract

**Background:**

Blood metagenomic next-generation sequencing, commercially known as the Karius test (Karius, Redwood City, CA), is a diagnostic tool that can aid clinicians in uncovering pathogens that might otherwise be challenging to diagnose by standard microbiological techniques. However, due to its cost, use in clinical practice is limited. Published studies have found conflicting impact on clinical management, thus the appropriate clinical scenarios for Karius testing require further investigation. We evaluated the indications for Karius testing and impact on antimicrobial management to develop evidence-based recommendations for test ordering at our institution.

**Figure:**
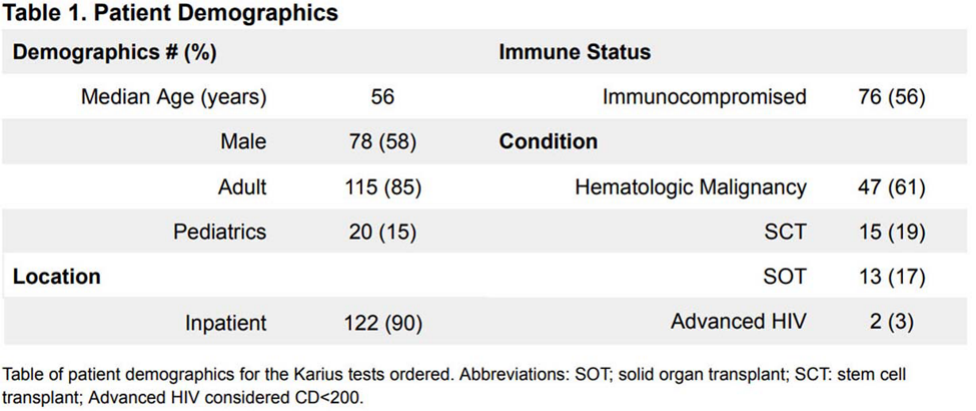

**Figure:**
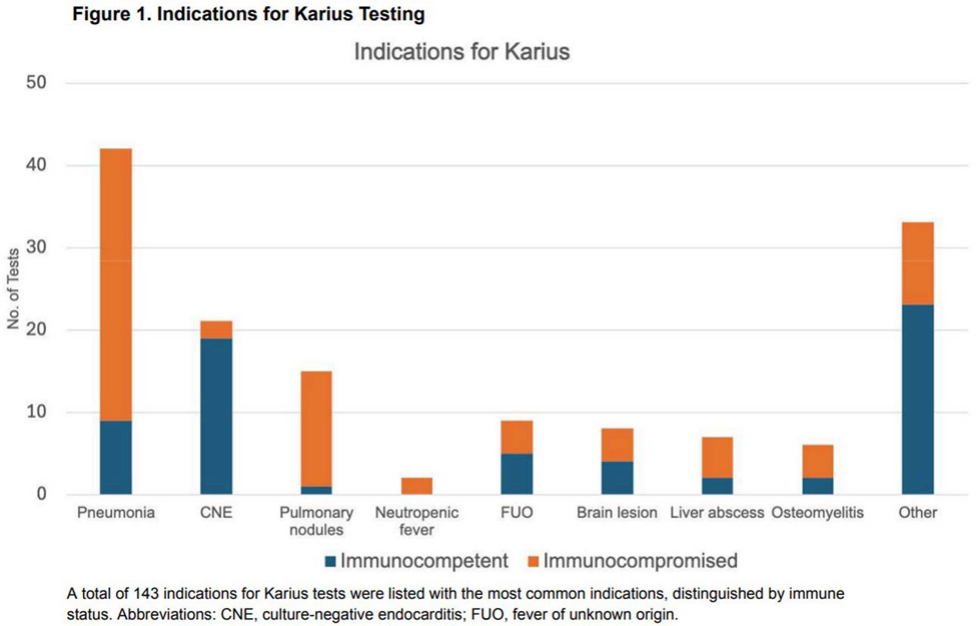

**Methods:**

In a retrospective chart review, we examined 135 Karius tests ordered between 2023-2024 at an academic medical center for pediatric and adult patients. Patient demographics and diagnoses associated with the test were collected. All microbiology and antimicrobial data before and after test results were examined. The clinical outcome of 30-day mortality was evaluated.

**Figure:**
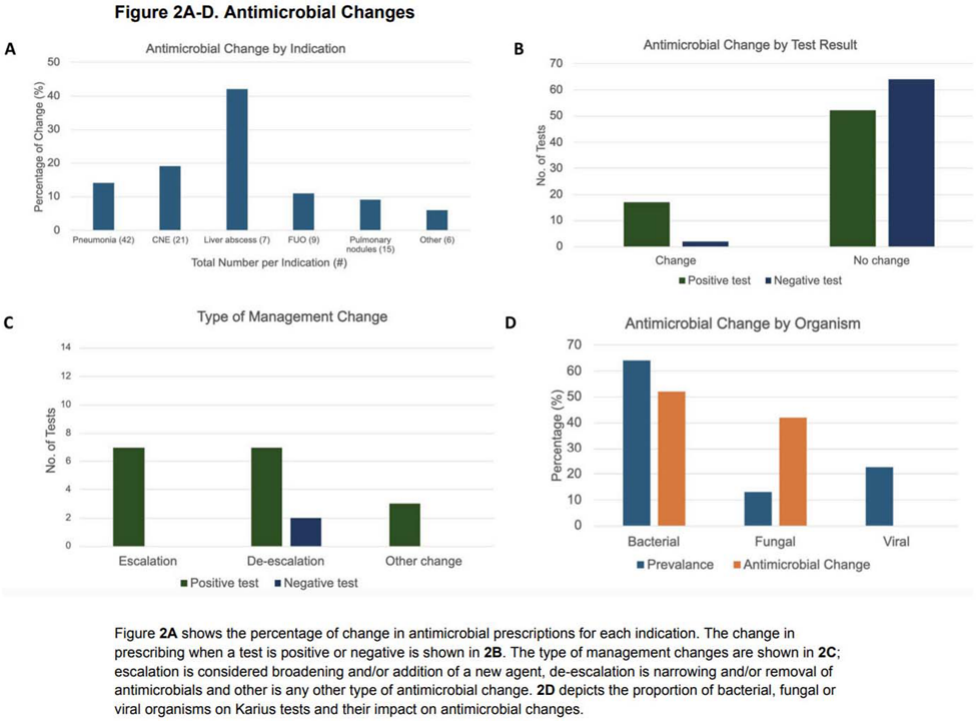

**Figure:**
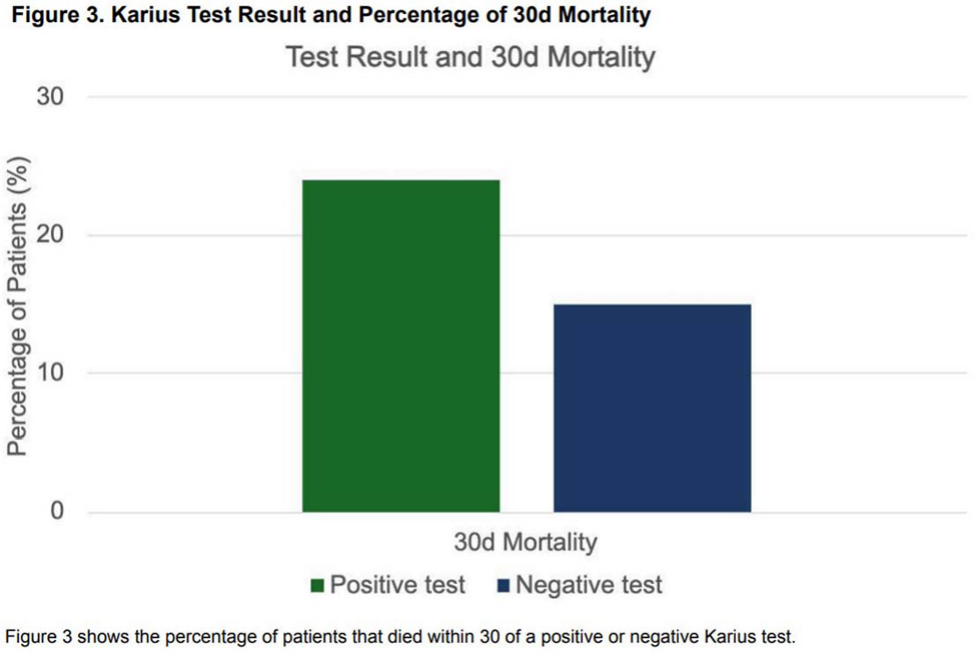

**Results:**

A total of 135 patients were tested, demographics shown in Table 1. There were 143 diagnoses listed as the indication for test ordering (Figure 1). There were 69 positive tests and 21% were concordant with microbiology cultures while 8% were discordant. There were 19 results (14%) that changed management. The diagnoses most likely to have management change in response to testing are shown in Figure 2A-B. The types of antimicrobial changes are listed in Figure 2C. A total of 142 organisms were identified, 91 bacterial, 32 viral and 19 fungal. Figure 2D depicts management changes occurring in bacterial (52%) and fungal organisms (42%). Within 30 days of the test, 20% patients died (Figure 3).

**Conclusion:**

In summary, we retrospectively assessed the indications and impact of Karius testing for patients at our institution. Karius tests changed antimicrobial management most often with liver abscess, culture-negative endocarditis, and pneumonia. While fungi represented a minority of organisms, they resulted in most of the antimicrobial changes. High rates of 30-day mortality were observed. Our data suggest Karius is most impactful with specific diagnoses, fungal pathogens, and non-critically ill patients.

**Disclosures:**

All Authors: No reported disclosures

